# An improved Scale-Invariant Feature Transform-based method for detecting tumor tissue motion induced by respiration

**DOI:** 10.1371/journal.pone.0330578

**Published:** 2026-06-24

**Authors:** Qingya Pan, Shengjie Luan, Fuke Zhang, Linzhao Tian, Guishu Wu, Yizhong Fan

**Affiliations:** Department of ChemoRadiotherapy Oncology, The Center Hospital of QingHe, HeBei, China; Majmaah University College of Applied Medical Sciences, SAUDI ARABIA

## Abstract

**Background:**

Scale-Invariant Feature Transform (SIFT) features are widely used in target recognition and tracking. This study aimed to exploit the robust performance of the SIFT algorithm to accurately calculate tumor tissue displacement during respiratory motion.

**Materials and methods:**

A thoracic phantom was employed in this study. Eight synthetic nodules with different shapes and gray values were inserted into the phantom. First, the nodules were displaced upward, downward, leftward, and rightward by 10 pixels to simulate motion in different directions during respiration. The nodules were then rotated clockwise and counterclockwise by 5°, 10°, 30°, and 45°. Subsequently, the SIFT and cross-correlation algorithms were applied to analyze the phantoms. Finally, a t-test was used to assess differences among motion directions.

**Results:**

The t-test result indicated no significant difference in the detection of moving phantom across all motion directions (p > 0.05). No statistically significant difference was observed between SIFT and cross-correlation in detecting translational motion. In contrast, the results obtained from rotating phantoms demonstrated that SIFT could effectively detect rotational distortion.

**Conclusion:**

The SIFT algorithm can be used to calculate tissue distortion caused by thoracic motion during respiration.

## 1. Introduction

Lung cancer is a disease with a high incidence and mortality rate. Nearly 70% of patients with cancer require radiotherapy during the course of treatment [1]. However, accurately determining tumor motion during respiration remains challenging. If tumor motion during breathing can be precisely quantified, radiation exposure to patients can be significantly reduced. At present, four-dimensional computed tomography (4DCT) is most commonly used in clinical practice to assess tumor motion; however, this approach is time-consuming and labor-intensive [2].

Previously, numerous studies have attempted to improve the accuracy of automatic motion detection. Emilie developed a tumor tracking technique capable of following a moving tumor in real time [3]. Christoph Hoog Antink used a high–sampling-rate terahertz homodyne spectroscopy system to estimate thoracic movement [4]. Evan et al. adapted the Microsoft Kinect v2 sensor to trace and record patient respiratory motion [5]. Mari Honda employed a breathing motion sensor for chest radiography during inspiration [6]. Melanie et al. used a self-gating 4D magnetic resonance imaging (MRI) workflow to assess the motion of the breast and organs at risk [7].

These studies have substantially improved the accuracy of tumor treatment and significantly reduced damage to surrounding organs at risk. However, chest tissue motion during respiration is not limited to simple translational displacement; a certain degree of rotational deformation also occurs with breathing. Owing to its invariance to rotation, the Scale-Invariant Feature Transform (SIFT) is widely used in target recognition and tracking [8,9], as well as in image segmentation and registration [10–12]. In this study, the robust performance of the SIFT algorithm was leveraged to accurately calculate tumor tissue displacement during respiratory motion. Both translational displacement and rotational angle were quantified to further enhance the accuracy of tissue motion assessment.

## 2. Materials and methods

**Fig 1:**
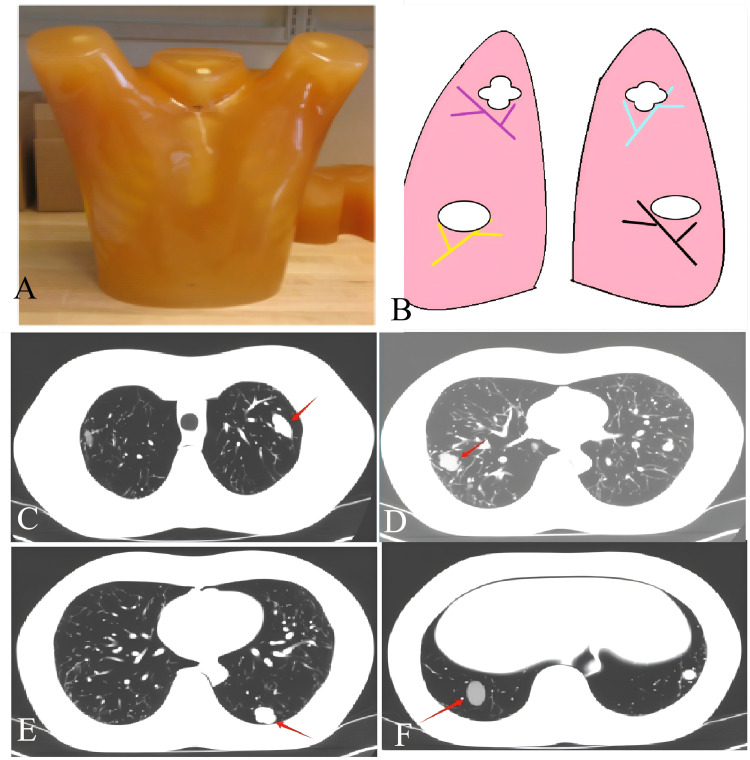
The thoracic phantom employed in this study and CT images. **A** The phantom **B** Schematic diagram of Nodule Layout. **C-F** are CT images for the nodules, **C** shows nodule 1, **D** shows nodule 2, **E** shows nodule 3, **F** shows nodule 4.

**Fig 2:**
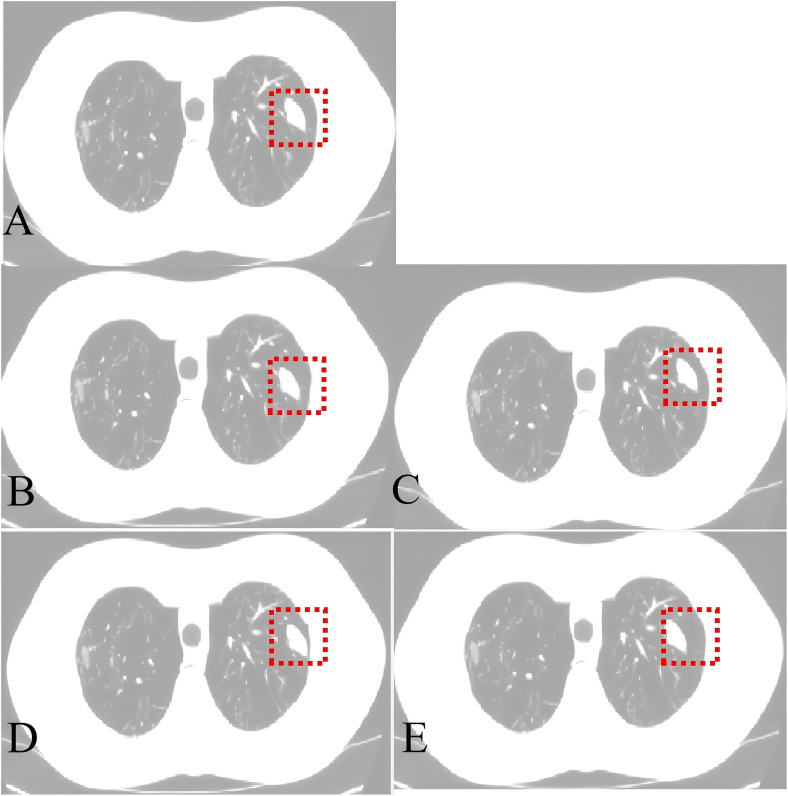
Shows the movement of the phantom (taking 2mL_up as an example). **(A)** original image of nodule, **(B)** nodule moves downward, **(C)** nodule moved upward, **(D)** nodule moves rightside, **(E)** nodule moves leftside.

**Fig 3:**
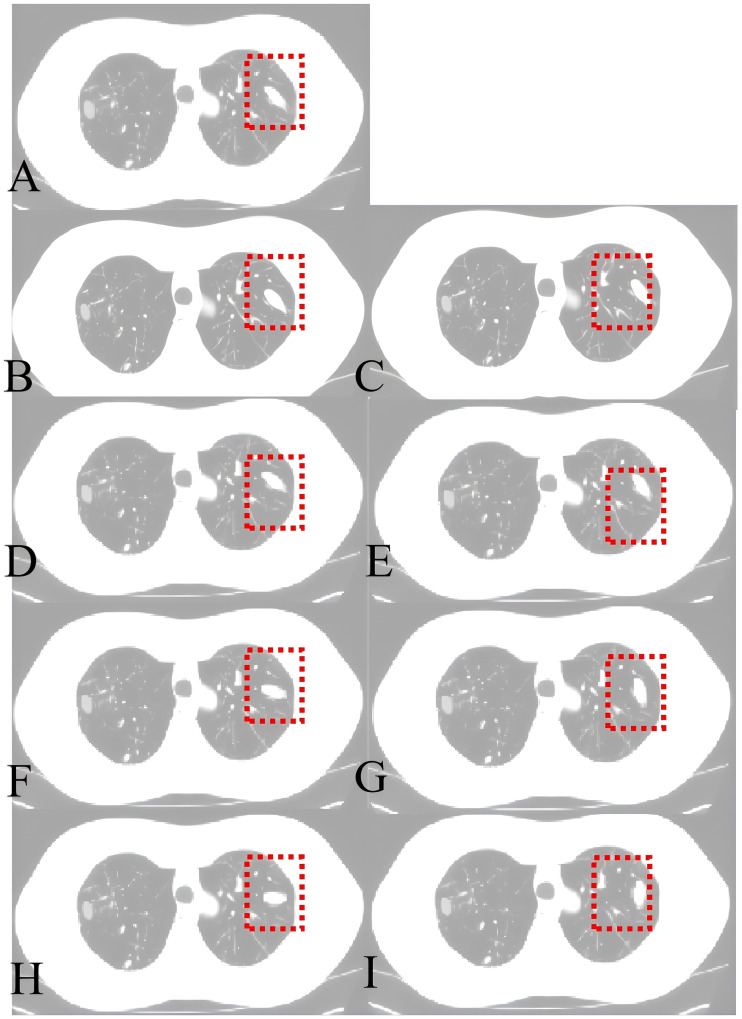
Shows the rotation of the phantom (taking 2mL_up as an example). **(A)** original image of nodule, **(B)** nodule rotate to left 5°, **(C)** nodule rotate to right 5°, **(D)** nodule rotate to left 30° **(E)** nodule rotate to left 30°, **(F)** nodule rotate to left 45.

The thoracic phantom employed in this study is shown in Fig 1. The phantom was scanned using a Philips 16-row CT scanner (Mx8000 IDT, Philips Healthcare, Andover, MA). Scans were acquired with a pitch of 1.00 and a slice thickness of 3.0 mm. Images were reconstructed with a slice thicknesses of 3.0 mm and a reconstruction increment of 1.5 mm. A total of eight synthetic nodules with different shapes and gray values were adopted in this experiment. The nodules included: (1) spherical, HU 100; (2) spherical, HU 100; (3) spherical, HU −630; (4) spherical, HU −630; (5) elliptical, HU 100; (6) lobulated, HU 100; (7) spiculated, HU −630; and (8) lobulated, HU −630. The nodules were displaced upward, downward, leftward, and rightward by 10 pixels to simulate translational motion, and the resulting configurations are shown in Table 1. The phantom position was repositioned between different nodule layouts. All CT images had a matrix size of 512 × 512 pixels for each slice [13]. Ten consecutive slices were analyzed for each nodule. Tumor position changes during respiration were simulated by moving the synthetic nodules by 10 pixels in each direction. This displacement was selected because the tumor motion range in this region is approximately 1 cm. The translated nodules are shown in Fig 2. In addition to translational motion, rotational motion was simulated by rotating the nodules by 5°, 10°, 15°, 30°, and 45°. The rotated nodules are shown in Fig 3. These rotation angles were selected because rib rotation during respiration typically ranges from 25° to 45°, whereas chest tissue rotation is generally smaller than that of the ribs.

**Table 1:** List of translated nodules and motion directions. Nodules 1-8 denote the identifiers of the synthetic nodules. For example: 1_up indicates that Nodule 1 was translated upward, 1_down indicates downward translation, 1_right indicates rightward translation, and 1_left indicates leftward translation.

Nodule Position	Nodule Motion Direction			
	Up	Down	Right	Left
Nodule 1	1_up	1_down	1_right	1_left
Nodule 2	2_up	2_down	2_right	2_left
Nodule 3	3_up	3_down	3_right	3_left
Nodule 4	4_up	4_down	4_right	4_left
Nodule 5	5_up	5_down	5_right	5_left
Nodule 6	6_up	6_down	6_right	6_left
Nodule 7	7_up	7_down	7_right	7_left
Nodule 8	8_up	8_down	8_right	8_left

A t-test was used to compare detection results among different movement directions. P < 0.05 was considered statistically significant.

### 2.1 SIFT

**Fig 4:**
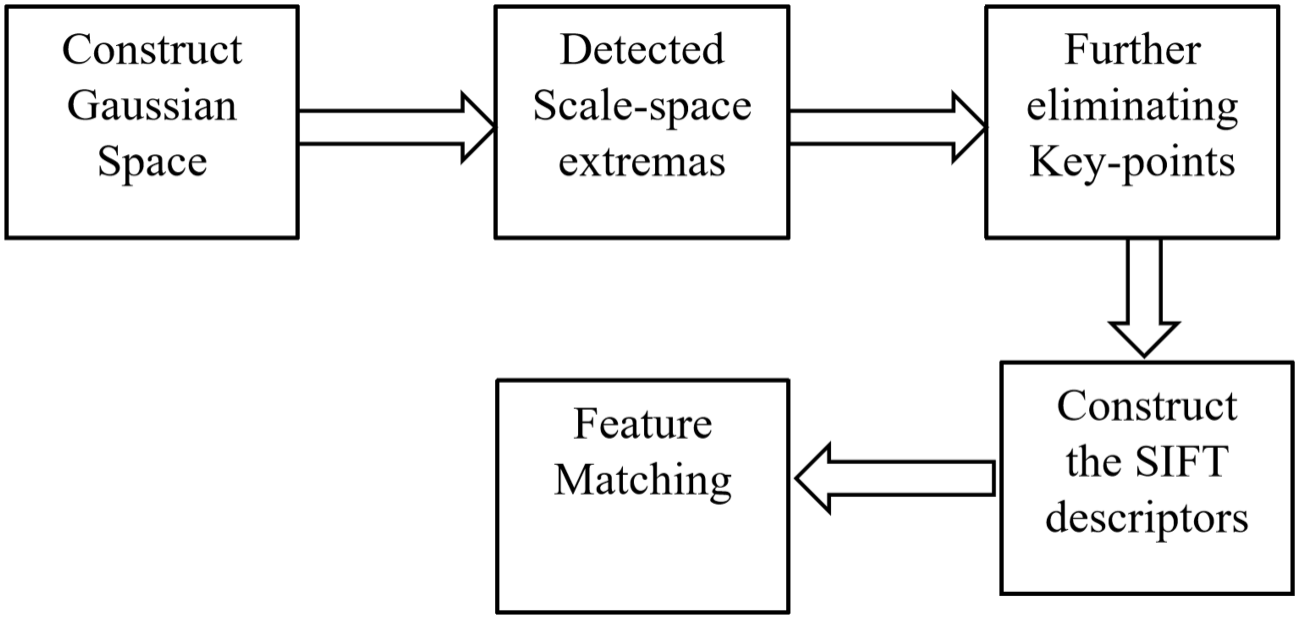
Flow chart of SIFT algorithm.

The SIFT is based on gradient orientation histograms constructed in Gaussian scale space. The overall calculation process is shown in Fig 4. The main steps of the algorithm are described as follows:

#### A. Gaussian scale-space construction.

Gaussian scale space is used to identify extreme points for the calculation of feature descriptors:


$$\begin{array}{c} \text{DG}\;(x,y,\sigma )=[G(x,y,n\sigma )-G(x,y,\sigma )]*I(x,y) \end{array}$$ (1)

where x and y denote the pixel coordinates of the image, I(x,y), G(x,y,σ) represents the Gaussian filter,  n = 1, 2, 3, 4, 5 denotes the scale coefficient, and DG(x,y,σ) represents the difference of Gaussian image. In this scale space, extrema are detected within the neighborhood of each point across the current, previous, and subsequent scale-space images.

In this study, the edge point of the known synthetic nodules were manually delineated. The contour points of the phantom nodules were then directly selected as candidate extrema for descriptor computation and feature matching.

#### B. Orientation assignment and descriptor generation.

The dominant orientation of each point is computed based on a gradient orientation histogram. The highest peak of the histogram is assigned as the main orientation of the key point.

Subsequently, a SIFT descriptor is constructed using the gradient magnitudes and orientations in the neighborhood of the detected key point. The scale of the key points is used to determine the radius of the search region. Gradient orientations are rotated according to the main orientation of the key point, and gradient histograms are then constructed around the key point. As a result, a 128-dimensional descriptor is obtained. In this study, a 30 × 30 pixel neighborhood around each key point was analyzed to identify the corresponding point in the translated phantom.

#### C. Feature matching.

The Euclidean distance was used to match the SIFT descriptors. First, the distances between all descriptor pairs were computed. Then, for each descriptor, the two closest descriptors were identified.

### 2.2 Cross-correlation

The cross-correlation function is a measure of similarity between two images. It calculates the cross-correlation in either the spatial or frequency domain to determine image movement in the spatial domain. In this study, the native MATLAB functions xcorr2 was used to compute 2D cross-correlation based on the dot-product method (Eq. 2), where the inputs are matrices.


$$\begin{array}{c} \gamma \;(u,v)=\frac{\sum_{x,y}[f(x,y)-{\overset{-}{f}}_{u,v}][t(x-u,y-v)-\overset{-}{t}]}{\{\sum_{x,y}[f(x,y)-{\overset{-}{f}}_{u,v}{]}^{\text{2}}\sum [t(x-u,y-v)-\overset{-}{t}{]}^{\text{2}}{\}}^{\text{0}.\text{5}}} \end{array}$$ (2)

where f denotes the original image, t denotes the template, $\overset{-}{t\;}$ represents the mean of the template, and $\;{\overset{-}{f}}_{u,v}\;$ represents the mean of the template of f(x,y).

Because nodules 1–4 were spherical, no difference was observed before and after rotation. The images of translated nodules are shown in Fig 2. Nodules 5–8 were rotated, and the corresponding images are shown in Fig 3. The parameters of the rotated nodules are summarized in Table 2. Both the SIFT and cross-correlation algorithms were independently applied to detect these nodules.

**Table 2:** List of rotated nodules and rotation angles. Nodules 5-8 denotes the identifiers of the synthetic nodules used for rotation experiments. For example, 5_L5 indicates that Nodule 5 rotated 5° to the left, whereas 5_R5 indicates Nodule 5 rotated 5° to the right.

	Rotated Nodule Position			
Rotation Angle	Nodule 5	Nodule 6	Nodule 7	Nodule 8
L5	5_L5	6_L5	7_L5	8_L5
L10	5_L10	6_L10	7_L10	8_L10
L30	5_L30	6_L30	7_L30	8_L30
L45	5_L45	6_L45	7_L45	8_L45
R5	5_R5	6_R5	7_R5	8_R5
R10	5_R10	6_R10	7_R10	8_R10
R30	5_R30	6_R30	7_R30	8_R30
R45	5_R45	6_R45	7_R45	8_R45

### 2.3 Evaluation criteria

The matching rate (MR) of two corresponding algorithms was calculated to evaluate the accuracy of the two algorithms and is defined as follows:


$$\begin{array}{c} \text{MR}=\frac{\text{Matc}{\text{h}}_{\text{num}}\;}{\text{min}\;(\text{N1},\;\text{N2})} \end{array}$$ (3)

where N1 denotes the number of detected points in image I1, N2 denotes the number of detected points in image I2, and Match_num_ represents the number of matched points.

All programs were developed using MATLAB 2024a (The MathWorks, Inc., Natick, MA, USA). Statistical analysis were performed using SPSS software, version 20.0 (IBM Corp., Armonk, NY, USA).

### 2.4 Ethics

This study is approved by the ethics committee of Qinghe Central Hospital.

## 3. Results

**Fig 5:**
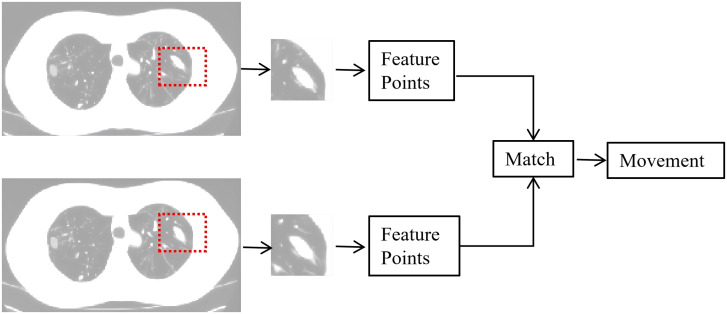
Flow chart of SIFT algorithm in this study.

**Fig 6:**
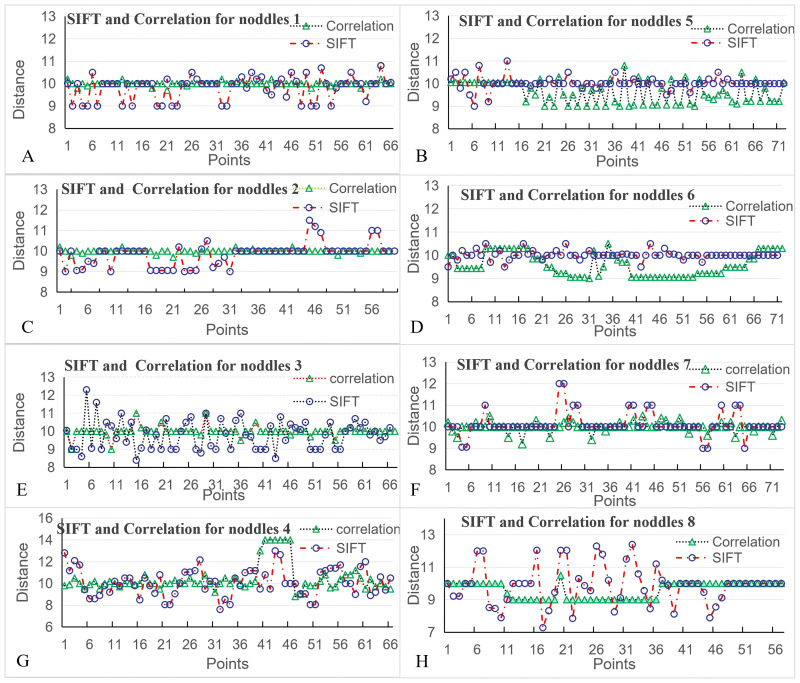
Presents a comparison between the SIFT and cross-correlation detection results across eight nodules.

**Fig 7:**
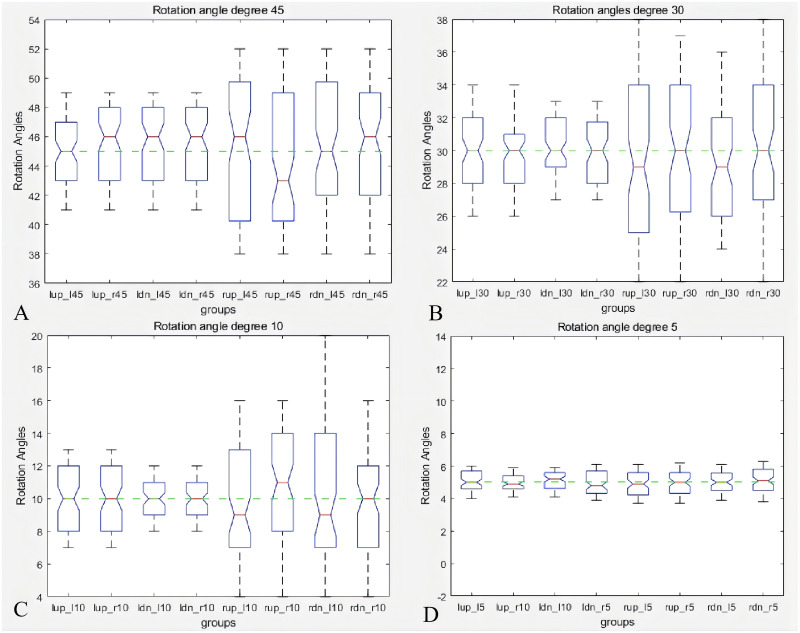
The detection results of the rotating nodule at various angular positions. **A** shows comparision of 45°, **B** shows comparision of 30°, **C** shows comparision of 10°, **D** shows comparision of 5°.

The statistical results of this experiment are summarized in Table 3. Table 3 illustrates the influence of different motion directions on the detection performance of the cross-correlation and SIFT algorithms. Most t-test results showed P < 0.05, indicating that no statistically significant differences were observed among the detection results of moving nodules in different directions. The t-test results for nodules showed no statistically significant differences for either the SIFT or cross-correlation algorithms (Nodule 5, rightward motion, P = 0.128; Nodule 6 downward motion, P = 0.312; Nodule 7, leftward motion, P = 0.534), indicating comparable detection performance across these movement conditions  Fig 5 and 6. Fig 7 presents a comparison of detection results between the SIFT and cross-correlation algorithms across eight synthetic nodules. Fig 7 shows box plots of the detection results for rotating nodules at various angular positions. The evaluated nodules included Nodule 5 (elliptical, HU 100), Nodule 6 (lobulated, HU 100), Nodule 7 (spiculated, HU −630), and Nodule 8 (lobulated, HU −630).

**Table 3:** t-test results for translated Nodules. (P < 0.05 was considered statistically significant).

Nodules	Correlation (p)			SIFT (p)		
	Down	Right	Left	Down	Right	Left
1	0.52	0.65	0.32	0.54	0.64	0.71
2	0.46	0.35	0.28	0.71	0.68	0.18
3	0.87	0.62	0.41	0.37	0.54	0.27
4	0.25	0.28	0.38	0.71	0.52	0.46
5	0.64	0.03	0.37	0.28	0.03	0.37
6	0.04	0.19	0.62	0.02	0.51	0.37
7	0.61	0.35	0.01	0.61	0.41	0.01
8	0.34	0.43	0.45	0.42	0.62	0.38

The MR was calculated for all eight nodules. The MR was determined using all detected feature points across 10 CT slices for each nodule. In Table 4, the measurement data are presented as mean ± standard deviation (x ± s).

**Table 4:** Matching rate results for eight synthetic nodules.

Nodules	Correlation	SIFT
1	0.90 ± 0.20	0.90 ± 0.15
2	0.89 ± 0.22	0.91 ± 0.18
3	0.88 ± 0.28	0.89 ± 0.20
4	0.86 ± 0.19	0.88 ± 0.17
5	0.95 ± 0.15	0.90 ± 0.20
6	0.86 ± 0.27	0.89 ± 0.19
7	0.88 ± 0.17	0.87 ± 0.25
8	0.85 ± 0.23	0.86 ± 0.24

## 4. Discussion

The SIFT algorithm has been widely applied across multiple fields, with numerous studies reporting significant results [14–19]. In this study, the SIFT algorithm was applied to calculate chest tissue motion during respiration. Unlike previous approaches, which primarily focused on translational displacement, the present method additionally accounts for rotational distortion. By leveraging the rotation invariance of the SIFT algorithm, distorted changes in chest tissue motion during respiration were quantitatively assessed.

Table 3 presents the t-test results for different movement directions. Downward, rightward, and leftward translations were compared with upward translation. As shown in Table 3, for most nodules and movement directions, no statistically significant differences were observed in the detection results. However, statistically significant differences were identified in a limited number of cases for specific nodules and directions. Further inspection of the phantom images revealed that, during motion, some Nodules overlapped with rib structures, resulting in severe boundary blurring. This overlap likely contributed to inaccurate detection results.

As shown in Fig 6, under a translational displacement of 10 pixels, the detected motion distances for all nodules fluctuated around the expected value of 10 pixels. The fluctuation observed for Nodules 1, 2, 5, and 6 were significantly smaller than those for Nodules 3, 4, 7, and 8, indicating higher detection accuracy for Nodules 1, 2, 5, and 6. This difference can be attributed to their relatively higher gray values (HU 100), which resulted in greater contrast with the surrounding tissues. During the calculation process, conversion to a grayscale image was performed, which resulted in a significant difference in nodule gray values. The cross-correlation detection results for Nodules 1–4 are also superior to those for Nodules 5–8. This is likely because Nodules 1–4 are circular, and their boundaries undergo minimal deformation during motion, resulting in more accurate detection.

The detection results obtained using the SIFT algorithm exhibited greater fluctuations compared to cross-correlation. This is because the key point filtering step was omitted to ensure accurate determination of all key-point positions. The calculation of all key points was performed to capture the known edges of the selected synthetic nodule. As shown in Fig 7, the SIFT algorithm was able to compute the rotation angles of Nodules 5–8. For right-side synthetic nodules with relatively low gray values (HU −630), such as Nodules 7 and 8, the calculated results fluctuated more. In contrast, left-side synthetic nodules with higher gray value (HU 100) exhibited smaller fluctuations. Future work may improve stability by optimizing this aspect of the calculation.

In addition, smaller rotation angles were associated with relatively low errors. This is primarily because the rotation angle influences changes in gray values around the nodules. Larger rotation angles result in greater changes around the nodules, which increase the likelihood of error during detection.

Another important consideration is that neither the SIFT algorithm nor the cross-correlation algorithm provides satisfactory detection results in uniform media. Detection in such regions requires further development of specialized algorithms. In this study, thoracic tissue motion was simplified to translational and rotational components, which imposes certain limitations for future clinical applications. Because the model does not represent biological tissue, the algorithm may not capture all possible complexities of real tissue movement. Advanced imaging techniques, such as 4DMRI, may offer improved accuracy in these scenarios.

## 5. Conclusion

This study quantified both movement and rotation of a thoracic phantom and demonstrated that the SIFT algorithm can be used to calculate distortion caused by thoracic tissue during respiratory motion. Although the method showed promising performance, the accuracy of rotation angle estimation still requires further improvement.
